# Neurological Response to cART vs. cART plus Integrase Inhibitor and CCR5 Antagonist Initiated during Acute HIV

**DOI:** 10.1371/journal.pone.0142600

**Published:** 2015-11-10

**Authors:** Victor G. Valcour, Serena S. Spudich, Napapon Sailasuta, Nittaya Phanuphak, Sukalaya Lerdlum, James L. K. Fletcher, Eugene D. M. B. Kroon, Linda L. Jagodzinski, Isabel E. Allen, Collin L. Adams, Peeriya Prueksakaew, Bonnie M. Slike, Joanna M. Hellmuth, Jerome H. Kim, Jintanat Ananworanich, SEARCH 010/RV 254 Study Group

**Affiliations:** 1 Department of Neurology, University of California San Francisco, San Francisco, California, United States of America; 2 Department of Neurology, Yale University, New Haven, Connecticut, United States of America; 3 Huntington Medical Research Institutes, Pasadena, California, United States of America; 4 South East Asia Research Collaboration with Hawaii, The Thai Red Cross AIDS Research Centre, Bangkok, Thailand; 5 Faculty of Medicine, Chulalongkorn University, Bangkok, Thailand; 6 Department of Retrovirology, Armed Forces Research Institute of Medical Sciences, United States Component, Bangkok, Thailand; 7 United States Military HIV Research Program, Walter Reed Army Institute of Research, Silver Spring, Maryland, United States of America; 8 Department of Biostatistics and Epidemiology, University of California San Francisco, San Francisco, California, United States of America; 9 Henry M. Jackson Foundation for the Advancement of Military Medicine, Bethesda, Maryland, United States of America; Pacific Northwest National Laboratory, UNITED STATES

## Abstract

**Objective:**

To compare central nervous system (CNS) outcomes in participants treated during acute HIV infection with standard combination antiretroviral therapy (cART) vs. cART plus integrase inhibitor and CCR5 antagonist (cART+).

**Design:**

24-week randomized open-label prospective evaluation.

**Method:**

Participants were evaluated then randomized to initiate cART (efavirenz, tenofovir, and either emtricitabine or lamivudine) vs. cART+ (cART plus raltegravir and maraviroc) during acute HIV and re-evaluated at 4, 12 and 24 weeks. We examined plasma and CSF cytokines, HIV RNA levels, neurological and neuropsychological findings, and brain MRS across groups and compared to healthy controls.

**Results:**

At baseline, 62 participants were in Fiebig stages I-V. Randomized groups were similar for mean age (27 vs. 25, *p* = 0.137), gender (each 94% male), plasma log_10_ HIV RNA (5.4 vs. 5.6, *p* = 0.382), CSF log_10_ HIV RNA (2.35 vs. 3.31, *p* = 0.561), and estimated duration of HIV (18 vs. 17 days, *p* = 0.546). Randomized arms did not differ at 24 weeks by any CNS outcome. Combining arms, all measures concurrent with antiretroviral treatment improved, for example, neuropsychological testing (mean NPZ-4 of -0.408 vs. 0.245, *p*<0.001) and inflammatory markers by MRS (e.g. mean frontal white matter (FWM) choline of 2.92 vs. 2.84, *p* = 0.045) at baseline and week 24, respectively. Plasma neopterin (*p*<0.001) and interferon gamma-induced protein 10 (IP-10) (*p* = 0.007) remained elevated in participants compared to controls but no statistically significant differences were seen in CSF cytokines compared to controls, despite individual variability among the HIV-infected group.

**Conclusions:**

A 24-week course of cART+ improved CNS related outcomes, but was not associated with measurable differences compared to standard cART.

## Introduction

The earliest events in Human Immunodeficiency Virus (HIV) infection, especially attendant innate and adaptive immune responses, are important to the understanding early steps related to neurological outcomes and viral reservoirs. HIV has been identified in cerebrospinal fluid (CSF) within weeks after infection and is associated with CNS immune activation and inflammation measured by brain imaging and CSF examination [1]. In the absence of early treatment, alterations in blood brain barrier (BBB) integrity as measured by plasma-CSF albumin ratio, and elevated CSF activation markers are noted within the first year prior among individuals not on antiretroviral therapy (ART) [2].

Early treatment may be protective. Neurofilament light chain (NFL), a marker of neuronal injury, is elevated during primary (up to one year post exposure) but not acute HIV infection [3, 4]. In primary HIV, NFL correlates to an array of toxic markers including CSF neopterin and interferon gamma-induced protein 10 (IP-10) [3]. Whether additional benefit is seen when standard combination ART (cART) is intensified with an integrase inhibitor and a CCR5 antagonist during acute HIV is not known [2]. Most publications related to cART intensification strategies have focused on chronic HIV stages and typically evaluate only systemic outcomes.

CCR5 antagonists are particularly important to examine during early infection because they have been shown to block entry of virus into cells with CCR5 receptors, including monocytes, a cell type that is tightly linked to CNS outcomes [5, 6]. In one macaque model study (n = 6), treatment with maraviroc, a CCR5 antagonist, was associated with reduced brain SIV RNA, DNA, and monocyte activation markers when compared to 22 historic untreated controls [7]. *In vitro* studies demonstrate inhibition of monocyte chemotaxis in response to a CCR5 antagonist, providing an additional potential mechanism for neuroprotection [8].

Clinical CNS benefits have been demonstrated in pilot studies of CCR5 antagonists when initiated during chronic HIV. A small case series of six individuals with neurological symptoms noted improvement in 5 out of 6 individuals whose treatment was intensified with maraviroc [9]. During chronic infection, HIV DNA burden in peripheral mononuclear cells (PBMC) enriched with CD14+ (i.e., monocytes) has been linked to HIV-associated neurocognitive disorders (HAND) and brain inflammation, both of which decline with maraviroc intensification [6, 10]. In this single arm study of maraviroc intensification (n = 12), participants experienced concurrent neuropsychological testing improvement.

Raltegravir blocks the pre-integration complex’s ability to bind to host DNA, resulting in a nonintegrated proviral HIV DNA that is rendered inactive [11]. One potential benefit of adding an integrase inhibitor to cART relates to maximizing the suppression of viral replication and reducing immune activation including T-cell activation [12, 13]. A small randomized study of raltegravir intensification failed to identify benefit on CSF immune activation markers or HIV RNA; however, participants had low levels of these disease markers at enrollment. A pooled investigation (n = 453) noted raltegravir-associated CNS toxicities in 10% of participants and linked these toxicities to concomitant use of medications that increase raltegravir blood levels [14]. Both maraviroc and raltegravir have moderate-to-good CNS penetration effectiveness (CPE) ratings and are therefore likely active in CNS during these studies [15, 16].

The current study was designed to investigate CNS-relevant outcomes of cART intensification when initiated during the earliest stages of acute HIV-1 infection, Fiebig stages I-V [17, 18]. Our study was embedded in a randomized cART-intensification scheme completed in a parent protocol with aims to determine factors that may inform peripheral reservoir establishment and cure strategies.

## Materials and Methods

### Selection of participants

This study evaluated 62 individuals consecutively enrolled between February 2010 and April 2012 who underwent randomization and were at least 24 weeks post randomization at the time of data analyses (NCT00796146, www.clinicaltrials.gov). As previously described, participants were identified from a voluntary counseling and testing (VCT) clinic at the Thai Red Cross AIDS Research Centre. [1, 18] All were confirmed to have acute HIV (Fiebig stages I-V). One participant with marked CSF pleocytosis and a positive serum syphilis test (i.e. possible neurosyphilis) was excluded from this analysis. HIV-uninfected Thai participants of similar age and gender underwent one-time neurological characterization including brain imaging (n = 28) and CSF sampling (n = 18) using the same processing algorithms and MRI/MRS prescription applied to HIV-infected participants. Normative neuropsychological data were available from over 500 healthy Thais. [19]

Neurological characterization occurred at baseline (i.e., week 0, pre-cART) and included MRI/MRS, lumbar puncture (LP), neurological examination, neuropsychological testing, and sampling for plasma markers. Optional CSF sampling was conducted for 27 and 22 participants at 0 weeks and 24 weeks, respectively. All other measures were repeated at 4, 12, and 24 weeks. The expedited pre-cART evaluations were typically completed within 48 hours; thus, more than 95% of cases initiated randomized therapy within three days of identification.

All participants signed written informed consents approved by the Institutional Review Boards at the University of California San Francisco (UCSF), Chulalongkorn University, Yale, and the Walter Reed Army Institute of Research (WRAIR). These same Institutional Review Boards also approved this study. Patient records and information were anonymized and de-identified prior to analysis.

### Intervention

Initial treatment consisted of efavirenz (EFV), tenofovir (TDF), and emtricitabine (FTC) in both arms with lamivudine (3TC) used interchangeably for FTC depending on availability. Raltegravir (RAL) and maraviroc (MVC) were added for the cART+ arm. In the cART only arm, participants who were intolerant to EFV (n = 2) or who had EFV resistance (n = 1) were switched to RAL whereas EFV was simply discontinued in the cART+ arm for those intolerant (n = 6) or resistant (n = 1). There were no MVC reassignments in either group.

### Neuropsychological characterization

Trained nurse-psychometrists conducted a four-item neuropsychological testing battery using a subset of our larger international HIV battery, as previously described [20]. The battery consisted of the Color Trails I and II, the Grooved Pegboard for the non-dominant hand, and the Trail-Making Test A. We calculated z-scores (standard deviations compared to Thai normative data) using standard techniques and defined an NPZ-4 as the arithmetic mean of the four z-scores from individual tests.

### Neurological examination and imaging

Participants underwent a standardized neurological examination by a trained clinician. We completed single-voxel MRS using a double spin echo data acquisition (PROBE-P) with TE = 35ms and TR = 1.5s on a GE Sigma HDx 1.5T scanner (GE Healthcare, Milwaukee, WI, software version 12-M4) using an 8-channel head coil for data reception and a standard body coil for transmission [21]. One co-investigator (NS) quantified all metabolites that were captured at left frontal white matter (FWM, 8cc), midline frontal gray matter (FGM, 8cc), posterior gray matter (OGM, 8cc), and basal ganglia (BG, 12cc). After each acquisition, we performed short echo-time (TE = 35ms) single voxel MRS of a standard spectroscopy phantom (GE Healthcare) to assess scanner stability. We used the time domain linear combination fitting software, LCModel (version 6.2, http://s-provencher.com/pages/lcmodel.shtml) for all metabolite quantification to measure N-acetyl aspartate (NAA), Creatine (Cr), Choline (Cho), myoinositol (MI), and glutamate and glutamine (Glx). Metabolites were analyzed as pure concentrations rather than ratios to Cr since descriptive analyses demonstrated a change in Cr longitudinally after initiation of antiretroviral treatment in each voxel (all p-values < 0.05), a finding also seen in chronic HIV (S1 Citation).

### Serum and CSF measurements

Although numerous cytokines were measured, to minimize type 1 error, our analyses were driven by *a priori* scientific hypotheses and focused on interleukin 6 (IL-6), IP-10, monocyte chemotactic protein 1 (MCP-1), and neopterin since these are tightly linked to CNS injury. We quantified IL-6 and MCP-1 in plasma and CSF by customized multiplex ELISA (Quansys Biosciences, Logan UT) captured on the Odyssey infrared imaging system (Li-Cor Biosciences, Lincoln, NE) and analyzed using Quansys Q-view Plus software (Quansys Biosciences). We employed traditional single-analyte ELISAs to measure IP-10 (Life Technologies, Grand Island NY) and neopterin (GenWay Biotech, San Diego, CA) and analyzed these with SoftMax Pro (Molecular Devices, Sunnyvale CA).

Blood CD4 T-lymphocyte subsets were measured by standard flow cytometry. The plasma HIV RNA level was measured by the Roche Amplicor HIV-1 Monitor Test v1.5 (Roche Diagnostics, Branchburg, NJ). The CSF HIV RNA level was measured in specimens that had been frozen for <6 months using a slight modification of the Roche Amplicor HIV-1 Monitor Test v1.5. RNA was extracted from ~200 μL of CSF by using the Boom silica extraction procedure (NucliSens Basic Isolation Reagents and Lysis buffer; bioMerieux, Durham, NC), as previously described [22]. The lower limit of detection was 50 copies/ml in plasma and 100 copies/ml in CSF. Neurofilament light chain was measured in CSF using a highly sensitive, two-site enzymatic quantitative immunoassay with a lower limit of detection of 50ng/L [3].

### Statistical considerations

We compared controls, cART, and cART+ groups at Week 0 using the Wilcoxon Rank Sum test for ordinal data, Chi-squared and Fisher’s Exact tests for categorical data, and Analysis of Variance (ANOVA) controlling for multiple comparisons for continuous data over the three groups over time. Paired t-tests and ANOVA were used to compare changes from baseline within groups over time. General Linear Models (GLM) controlling for baseline values were used to compare the two treatment groups, and when comparing the HIV group with the control group, covariates included age, gender, and mean educational level. Analyses of MRS data were examined using GLM and ANOVA controlling for multiple comparisons. Because of the novelty of this work and the small sample size, actual p-values are presented. For interpretation, we recommend a false discover rate proposed by Benjamini for clinical data, which, for four tests within each voxel, gives a p-value of 0.028 as the cutoff for significance [23]. Due to floor effects for IL-6 in plasma and CSF, we did not complete statistical analyses for this cytokine. All statistical analyses were performed in Stata v13.2. Figures were produced with Prism 6.0. An overall p-value of 0.05 (corrected for multiple comparisons, if necessary) was used for testing statistical significance.

## Results

### Baseline composition of the sample

We randomized 62 participants resulting in treatment arms that were well-matched on key demographic and clinical variables including age, gender, baseline CD4+ T-lymphocyte count, and both plasma and CSF HIV RNA level (Table 1, S1 Dataset). The HIV-uninfected Thai controls were more frequently female (*p*<0.001), were older (*p* = 0.007), and had lower total years of education (*p*<0.001) compared with the HIV-infected groups combined. No differences were seen between treatment groups in Fiebig stage (*p* = 0.842, Fisher’s exact test) comparing stages 1 & 2 grouped together and 3 & 4 & 5 grouped together. When all Fiebig stages in cART are compared with all stages in cART+ using Kendall’s tau-b chi-squared test, no differences between groups were seen (*p* = 0.463). There were three participants in the cART arm and seven participants in the cART+ arm who discontinued EFV (p = 0.326).

**Table 1:** Baseline characteristics of the two treatment arms and comparison of HIV-infected to HIV-uninfected controls.

	cART	cART+	Control	p-value cART vs. cART+	p-value HIV+ vs. HIV-
Enrollment, sample size	30	32	29		
Age, median (range) years	27 (18–47)	28 (20–49)	35 (22–46)	0.137^1^	0.007^1^
Gender, n (%) male	28 (93)	30 (94)	16 (55)	0.999^2^	<0.001^2^
Education, mean (SD) years	16 (3.4)	17 (3.6)	13 (5.4)	0.849^3^	<0.001^3^
Estimated duration of HIV [Number of days, mean (SD)]	18 (6.8)	17 (7.8)	—-	0.546^3^	n/a
Fiebig stage [n (%) at F1/F2 vs. F3/F4/F5]	14 (47%)	13 (41%)	—-	0.842^2^	n/a
Baseline CD4 T-lymphocyte count, median (IQR)	369 (277)	391 (218)	—	0.706^1^	n/a
Baseline Plasma viral load, mean log_10_ (SD)	5.5 (1.11)	5.6 (1.33)	—	0.838^3^	
Baseline CSF viral load, mean log_10_ (SD)	2.1 (1.89)	3.3 (1.57)		0.059^3^	

1.Wilcoxon Rank Sum test

2. Fisher’s Exact test

3. Student t-test.

### Clinical variables

After 24 weeks of treatment, 4 (12.5%) cART and 3 (9.4%) cART+ participants had detectable plasma HIV RNA and the mean (range) by group was 53 (50–102) and 234 (50–5596) copies/ml in cART and cART+ groups, respectively (*p* = 0.316, comparison of log_10_ HIV RNA, *p* = 0.406). CSF HIV RNA was below the level of detection of our assay in all participants who underwent repeat lumbar puncture (n = 7 cART and 12 cART+). When adjusted for baseline value, the change in CD4 T-lymphocyte count did not differ by treatment arm [mean (standard deviation, SD) of 238 (202) and 225 (243) for the cART and cART+ arms, respectively (*p* = 0.812)]. In a model predicting HIV RNA (log_10_) adjusted for CD4 T-lymphocyte count and time, there is a significant difference in the slope of cART+ vs. cART (*p* = 0.017, cART+ coefficient = -0.244) with significant coefficients for CD4 T-lymphocyte count and week (*p*<0.001).

### Neuropsychological outcomes

**Fig 1:**
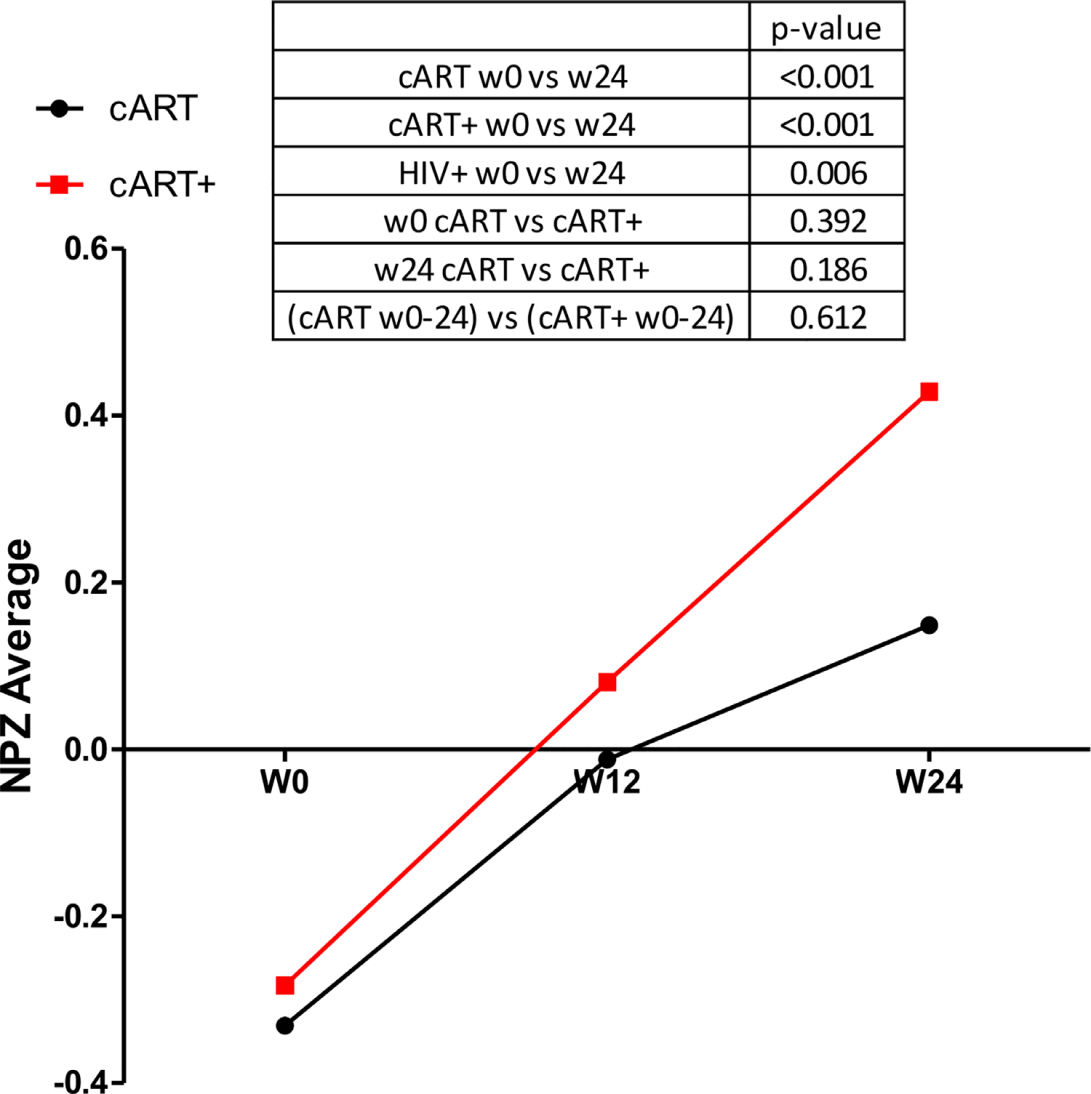
Change in neuropsychological testing performance (NPZ-4) over 24 weeks by randomized arm. Participants demonstrate improvement with no differences noted by arm.

Neuropsychological test performance improved in both arms (Fig 1). At baseline, the mean (SD) NPZ-4 score was -0.33 (0.82) and -0.28 (0.85) for the cART vs. cART+ groups, respectively (*p* = 0.392). The 24-week change in NPZ-4 did not differ by arm when adjusted for baseline level (0.59 and 0.69 for cART vs. cART+, *p* = 0.612). At 24 weeks, the mean (SD) NPZ-4 was 0.15 (0.96) and 0.42 (0.59) for the cART and cART+ groups, respectively (*p* = 0.139). Based on our sample size and assuming statistical significance at the 0.05 level, should differences exist by arm, they are likely smaller than 0.6 z-score units.

### Change in MRS

**Fig 2:**
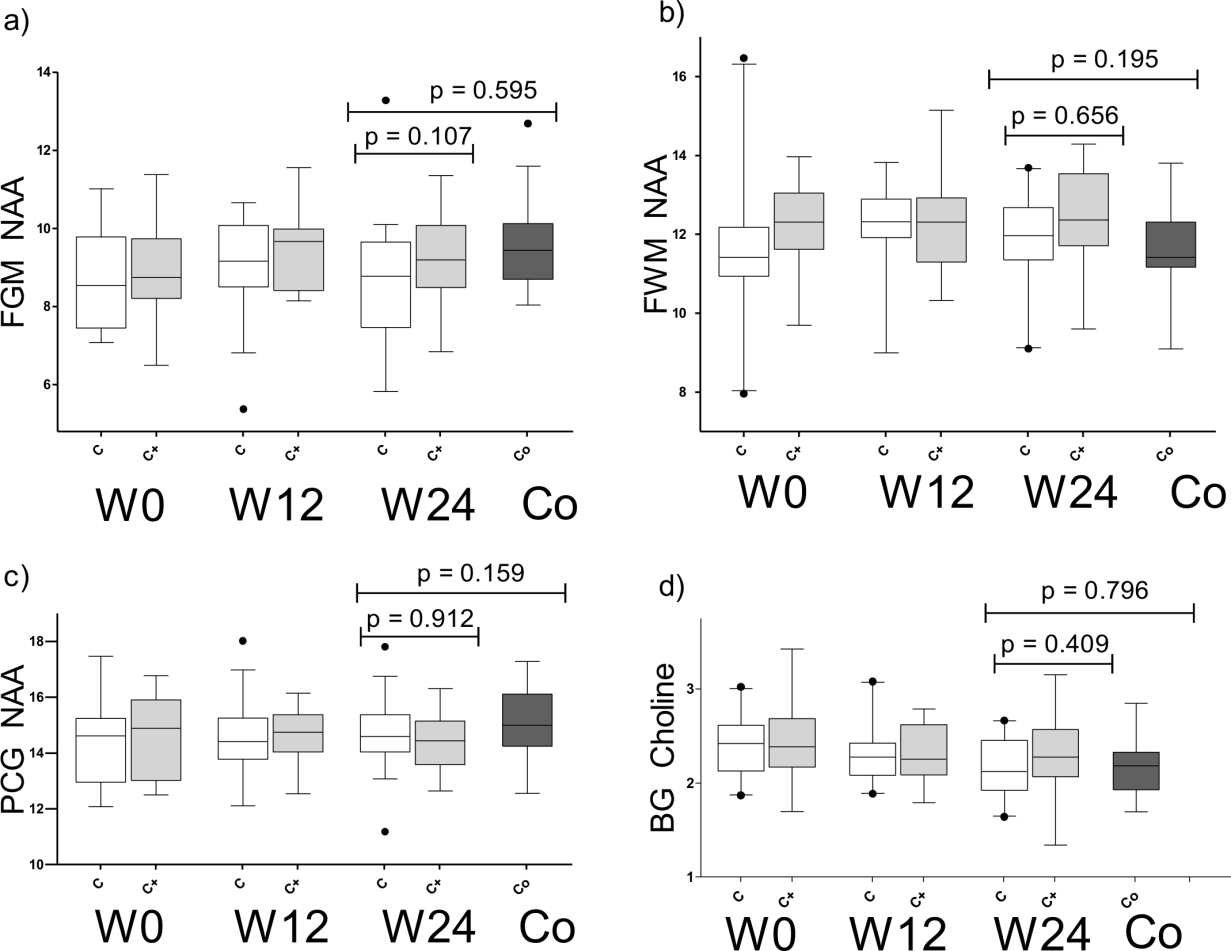
NAA and Cho changes with treatment cART (white) vs. cART+ (grey) compared to controls (black). (a) Frontal grey matter (b) Frontal white matter (c) Posterior cingulate gyrus (d) Basal ganglia.

Longitudinally, 44, 41, 41, and 42 participants completed the MRS at weeks 0, 4, 12, and 24, respectively. Improvements with treatment were statistically significant or approached significance in analyses of all HIV-infected participants (both arms combined) for NAA (FGM, FWM, PGM) and Cho (BG) in comparisons of week 0 with week 24 values (*p* = 0.044, *p* = 0.037, *p* = 0.046, and *p* = 0.023, respectively). No statistically significant changes were seen between groups or over time at weeks 4 and 12 and these results are not reported. We did not identify differences in these metabolites by study arm (Fig 2). Across all voxels, we noted a trend toward improvement in FWM Cho by study arm with the cART+ group trending toward a larger change, but this did not meet our threshold of significance (*p* = 0.047); and, this difference did not remain after adjustment for baseline measures (*p* = 0.210). Despite changes noted in NAA across multiple voxels, the level of NAA trended or remained lower at week 24 in pooled analyses among HIV-infected participants compared to controls (p-values: 0.032 for FGM NAA, 0.047 for FWM NAA, 0.028 for BG NAA, and 0.005 for PGM NAA). In contrast, BG Cho level declined to a level that was similar to controls at week 24 (*p* = 0.796). We did not identify differences in the change of any metabolite by randomized arm. Based on our sample size and at the 0.05 level of significance, we had 80% power to detect differences of 0.04 and 0.03 for FWM NAA and FWM Cho, respectively.

### Plasma and CSF inflammatory biomarkers

**Fig 3:**
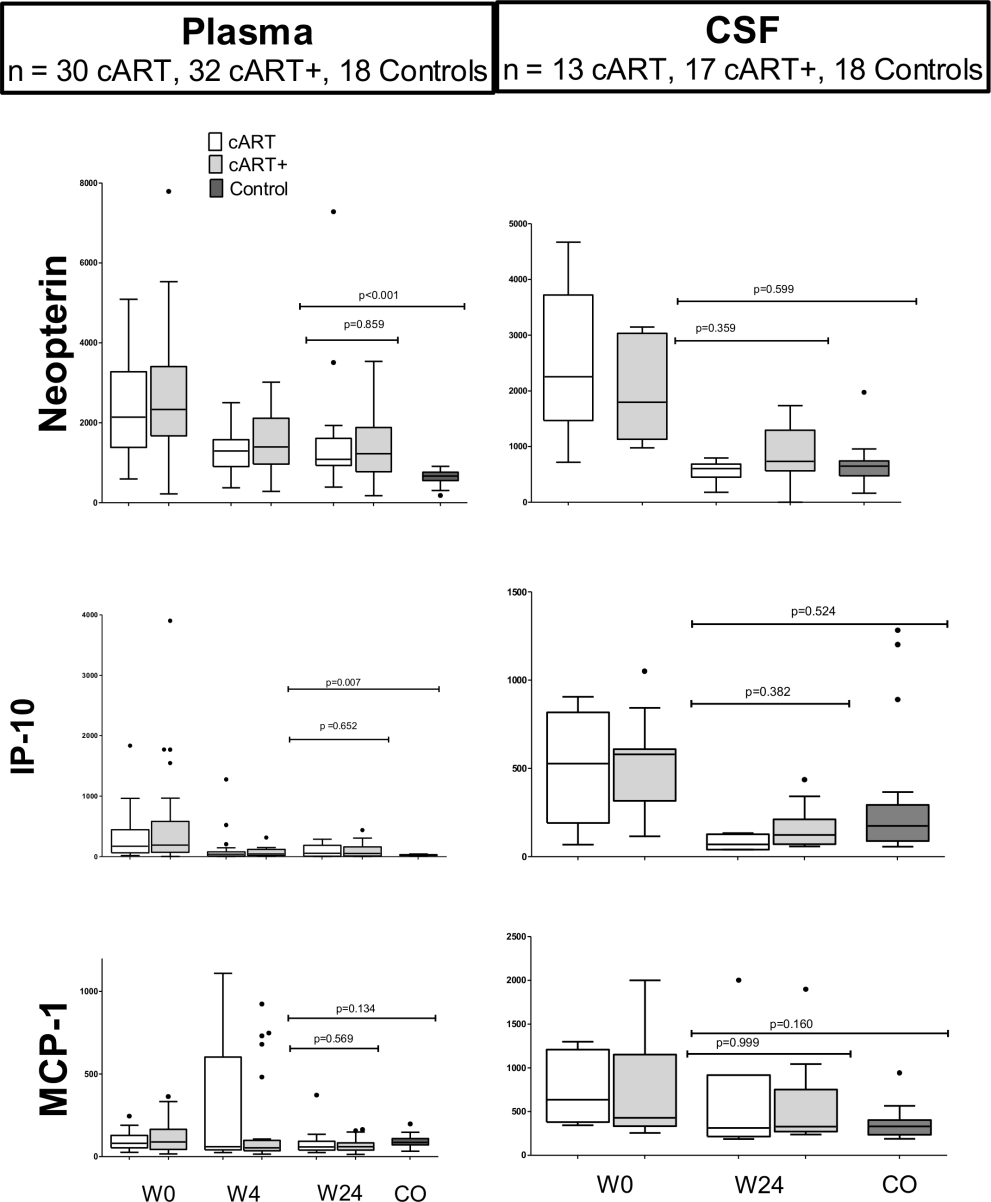
Tukey boxplots of cytokine changes in cART (white) vs. cART+ (grey) compared to controls (black). IL-6 not displayed because it was constitutively undetectable (controls) and seldom elevated in HIV, particularly in plasma. Neopterin and IP-10 remain elevated in plasma after 24 weeks on ART. CSF cytokines decrease with treatment and appear similar to controls.

Plasma cytokines were measured on 62, 60, 61, and 61 participants at weeks 0, 4, 12, and 24, respectively. Over 24 weeks, we identified a decrease after antiretroviral treatment for neopterin (*p*<0.001) and IP-10 (*p*<0.001) with a trend level decrease in MCP-1 (*p* = 0.061) when both arms were combined. The change in these cytokine levels did not differ across arms (*p*’s all >0.05, Fig 3). In univariate analyses of variance, the change in the cART+ group was significantly larger than that in the cART group at 4 weeks for neopterin (*p* = 0.002), IP-10 (*p* = 0.001) and MCP-1 (*p* = 0.013) but there was no difference between treatment groups at 24 weeks. Plasma levels of both IP-10 and neopterin remained higher than those of the Thai controls at week 24 (p’s<0.001).

Cytokines were measured in CSF on 27 and 22 participants at weeks 0 and 24, respectively. In general, we noted greater variability in CSF cytokines among HIV-infected participants compared to uninfected controls, suggesting heterogeneity where some participants had a less complete resolution of elevations than others. The coefficient of variation (SD/mean), measuring the variability around the mean for the CSF cytokines, ranged from 1% to 56% in the uninfected controls vs. 76% to 120% among the HIV-infected participants (*p* = 0.022). Although greater variability was noted among HIV-infected groups (n = 19), there were no statistically significant differences noted in CSF cytokines at 24 weeks compared to controls.

## Discussion

This work identifies broad improvements in neurological markers associated with ART initiated within days of HIV infection. However, we did not find differences in the arm where cART was augmented with an integrase inhibitor and CCR5 antagonist. This study did not include an arm of HIV-infected patients who do not start cART; thus, we are unable to estimate the amount of change attributable to therapy. However, there are data demonstrating detrimental effects as early as the first year of HIV in the absence of treatment [2, 24]. Together, our data and published work bolster the argument for early therapy; yet, the immediate clinical relevance is limited since identifying individuals to have HIV during seroconversion remains a challenging task.

This study leads to a number of other considerations relevant to CNS outcomes. Despite treatment of these individuals during the earliest stages of infection, cytokine that are pertinent to the CNS remain higher in the blood and MRS NAA remained lower than that of our controls. We measured these cytokines (MCP-1 and IP-10) because they are linked to monocyte function increasing the relevance to neuroHIV. We did not identify persistent abnormalities in CSF cytokines within a small sample nor did we note abnormalities on neuropsychological testing, which may require a longer duration of inflammation to develop. It is possible that demographic differences between controls and HIV-infected participants (i.e. gender, age, lifestyle issues) influenced this finding; however, we are unaware of data elsewhere to support the claim that elevations of these markers are associated with male gender or younger age, as seen in our HIV-infected group. A sensitivity analysis omitting three patients with cART resistance or intolerance did not change the overall results or statistical calculations.

This is the first attempt to look systematically at the CNS impact of intensification strategies implemented during acute HIV. Results from systemic studies are mixed. A small case series of ten participants with CSF HIV RNA suppressed to less than 2 copies did not identify changes in HIV RNA or neopterin associated with MVC intensification [25]. A 12-week, randomized, open-label pilot study of RAL intensification in chronic HIV patients with suppressed plasma HIV RNA did not identify a reduction in intrathecal immune or CSF HIV RNA [26].

This study focused on 6-month follow-up of cART treated AHI, and resolution of immune activation could require longer treatment. Since all cART+ subjects were switched to standard cART at 6 months, the impact of longer treatment with the cART+ regimen is unknown. It is possible that resolution of immune activation could require further treatment. We are also unable to determine from the current analysis if an initial 6-month intensification strategy is associated with improvement in long-term outcomes (e.g. beyond 6 months).

In sum, the present study identifies improvement in important makers concurrent with treatment during acute HIV and important residual abnormalities pertinent to CNS outcomes. Over this 24-week period, we did not identify incremental benefit associated with intensification with MVC and RAL. We cannot exclude the possibility that smaller differences would emerge with a larger sample of participants or more sensitive measures. Although intensification with MVC and RAL was not associated with improvement, small unresolved abnormalities that persist at 24 weeks raise the possibility for improvement beyond standard cART to reduce viral trafficking that is theorized to mechanistically drive neurological injury in later stages of disease.

## Supporting Information

S1 Citation Citation in press. (DOCX)

S1 Dataset Manuscript Dataset. (XLSX)
