# Supplementary material for: Neurological Response to cART vs. cART plus Integrase Inhibitor and CCR5 Antagonist Initiated during Acute HIV
Source: PLoS One. 2015 Nov 10;10(11):e0142600. doi: 10.1371/journal.pone.0142600 (PMC4640512; doi:10.1371/journal.pone.0142600)
Supplement: S1 Citation — (DOCX) [file pone.0142600.s001.docx]

**Supporting Information**

**S1 Citation, in press.** Sailasuta N, Ananworanich J, Lerdlum S, Sithinamsuwan P, Fletcher JL, Tipsuk S, et al. Neuronal-glia markers by Magnetic Resonance Spectroscopy in HIV Before and After Combination Antiretroviral Therapy. J Acquir Immune Defic Syndr.
